# Supplementary material for: An annotation-free whole-slide training approach to pathological classification of lung cancer types using deep learning
Source: Nat Commun. 2021 Feb 19;12:1193. doi: 10.1038/s41467-021-21467-y (PMC7896045; doi:10.1038/s41467-021-21467-y)
Supplement: Supplementary file 1 — Reporting Summary [file 41467_2021_21467_MOESM1_ESM.pdf]

nature research

Corresponding author(s): Chao-Yuan Yeh, Cheng-Yu Chen

Last updated by author(s): Jan 13, 2021

Reporting Summary

Nature Research wishes to improve the reproducibility of the work that we publish. This form provides structure for consistency and transparency in reporting. For further information on Nature Research policies, see our Editorial Policies and the Editorial Policy Checklist.

Statistics

For all statistical analyses, confirm that the following items are present in the figure legend, table legend, main text, or Methods section.

n/a	Confirmed	
☐	☒	The exact sample size (n) for each experimental group/condition, given as a discrete number and unit of measurement
☐	☒	A statement on whether measurements were taken from distinct samples or whether the same sample was measured repeatedly
☐	☒	The statistical test(s) used AND whether they are one- or two-sided<br>*Only common tests should be described solely by name; describe more complex techniques in the Methods section.*
☒	☐	A description of all covariates tested
☒	☐	A description of any assumptions or corrections, such as tests of normality and adjustment for multiple comparisons
☐	☒	A full description of the statistical parameters including central tendency (e.g. means) or other basic estimates (e.g. regression coefficient) AND variation (e.g. standard deviation) or associated estimates of uncertainty (e.g. confidence intervals)
☐	☒	For null hypothesis testing, the test statistic (e.g. F, t, r) with confidence intervals, effect sizes, degrees of freedom and P value noted<br>*Give P values as exact values whenever suitable.*
☒	☐	For Bayesian analysis, information on the choice of priors and Markov chain Monte Carlo settings
☒	☐	For hierarchical and complex designs, identification of the appropriate level for tests and full reporting of outcomes
☒	☐	Estimates of effect sizes (e.g. Cohen's d, Pearson's r), indicating how they were calculated

Our web collection on statistics for biologists contains articles on many of the points above.

Software and code

Policy information about availability of computer code

Data collection	Slide data were scanned by Hamamatsu NanoZoomer XR and opened by OpenSlide 3.4.1.
Data analysis	A custom code for data analysis is provided in https://github.com/aetherAI/whole-slide-cnn, requiring Tensorflow 1.15.3 and Horovod 0.19.0. In our experimental environment, CUDA 10.0 and cudNN 7.6 are installed for GPU acceleration, and Open MPI 4.0.1 and MPI for Python 3.0.3 are installed for distributed computation.

For manuscripts utilizing custom algorithms or software that are central to the research but not yet described in published literature, software must be made available to editors and reviewers. We strongly encourage code deposition in a community repository (e.g. GitHub). See the Nature Research guidelines for submitting code & software for further information.

Data

Policy information about availability of data

All manuscripts must include a data availability statement. This statement should provide the following information, where applicable:

- Accession codes, unique identifiers, or web links for publicly available datasets
- A list of figures that have associated raw data
- A description of any restrictions on data availability

The raw data of models' predictions, learning curves and throughputs are provided as Supplementary Data 1. The slide data from TMUH, WFH and SHH are not publicly available due to patient privacy constraints, but are available upon reasonable request from the corresponding author Chao-Yuan Yeh or Cheng-Yu Chen. The slide data supporting the cross-site generalization capability in this study are obtained from TCGA via the Genomic Data Commons Data Portal (https://gdc.cancer.gov).

Field-specific reporting

Please select the one below that is the best fit for your research. If you are not sure, read the appropriate sections before making your selection.

- [x] Life sciences
- [ ] Behavioural & social sciences
- [ ] Ecological, evolutionary & environmental sciences

For a reference copy of the document with all sections, see nature.com/documents/nr-reporting-summary-flat.pdf

Life sciences study design

All studies must disclose on these points even when the disclosure is negative.

Sample size	No sample-size calculation was performed. Within the period from 2018 to 2019, all slides of lung resection and biopsy collected from TMUH, WFH and SHH are included in this study.
Data exclusions	We exclude slides that are not diagnosed as adenocarcinoma, squamous cell carcinoma, or benign, since the current study focus on identifying these types.
Replication	Custom codes are provided for reproducing the results. (https://github.com/aetherAI/whole-slide-cnn, https://github.com/aetherAI/tensorflow-huge-model-support)
Randomization	Slide samples were randomized fairly into training, validation and testing datasets.
Blinding	No investigator blinding is necessary since our experiments are based on slide images and software analysis. The data are split randomly into 3 sets and the test set do not involved in any part of model tuning.

Reporting for specific materials, systems and methods

We require information from authors about some types of materials, experimental systems and methods used in many studies. Here, indicate whether each material, system or method listed is relevant to your study. If you are not sure if a list item applies to your research, read the appropriate section before selecting a response.

Materials & experimental systems

n/a	Involved in the study	
x		Antibodies
x		Eukaryotic cell lines
x		Palaeontology and archaeology
x		Animals and other organisms
	x	Human research participants
x		Clinical data
	x	Dual use research of concern

Methods

n/a	Involved in the study	
x		ChIP-seq
x		Flow cytometry
x		MRI-based neuroimaging

Human research participants

Policy information about studies involving human research participants

Population characteristics	Slide images of lung biopsy and resection from patients that were diagnosed at TMUH, SHH and WFH within 2018-2019.
Recruitment	This study did not involve participant recruitment.
Ethics oversight	The access of data was compliant with policies (NO.1080506_1) approved by the Office of Legal Affairs, Taipei Medical University and national legislation for research. The data collections were allowed proceeding after informed consents were obtained from participants. All WSIs were de-identified and do not contain any patient information or label text except for slide-level diagnosis. The collected data are limited to research use only.

Note that full information on the approval of the study protocol must also be provided in the manuscript.

Dual use research of concern

Policy information about dual use research of concern

Hazards

Could the accidental, deliberate or reckless misuse of agents or technologies generated in the work, or the application of information presented in the manuscript, pose a threat to:

No	Yes	
☒	☐	Public health
☒	☐	National security
☒	☐	Crops and/or livestock
☒	☐	Ecosystems
☒	☐	Any other significant area

Experiments of concern

Does the work involve any of these experiments of concern:

No	Yes	
☒	☐	Demonstrate how to render a vaccine ineffective
☒	☐	Confer resistance to therapeutically useful antibiotics or antiviral agents
☒	☐	Enhance the virulence of a pathogen or render a nonpathogen virulent
☒	☐	Increase transmissibility of a pathogen
☒	☐	Alter the host range of a pathogen
☒	☐	Enable evasion of diagnostic/detection modalities
☒	☐	Enable the weaponization of a biological agent or toxin
☒	☐	Any other potentially harmful combination of experiments and agents
