# Supplementary material for: An annotation-free whole-slide training approach to pathological classification of lung cancer types using deep learning
Source: Nat Commun. 2021 Feb 19;12:1193. doi: 10.1038/s41467-021-21467-y (PMC7896045; doi:10.1038/s41467-021-21467-y)
Supplement: Supplementary file 2 — Supplementary Information [file 41467_2021_21467_MOESM2_ESM.pdf]

An Annotation-free Whole-slide Training Approach to Pathological Classification of Lung Cancer Types by Deep Learning

Chi-Long Chen[1,2,3*], Chi-Chung Chen[4*], Wei-Hsiang Yu[4], Szu-Hua Chen[4], Yu-Chan Chang[5], Tai-I Hsu[6], Michael Hsiao[6], Chao-Yuan Yeh[4**], Cheng-Yu Chen[7,8**]

1 Department of Pathology, School of Medicine, College of Medicine, Taipei Medical University, Taipei, Taiwan

2 Department of Pathology, Taipei Medical University Hospital, Taipei, Taiwan

3 Research Center for Artificial Intelligence in Medicine, Taipei Medical University, Taipei, Taiwan

4 aetherAI, Co., Ltd., Taipei, Taiwan

5 Department of Biomedical Imaging and Radiological Sciences, National Yang-Ming University, Taipei, Taiwan

6 Genomics Research Center, Academia Sinica, Taipei, Taiwan

7 Department of Radiology, School of Medicine, College of Medicine, Taipei Medical University, Taipei Taiwan

8 Department of Radiology, Taipei Medical University Hospital, Taipei, Taiwan

Correspondences:

Cheng-Yu Chen, Department of Radiology, Taipei Medical University Hospital, 252, WuXing Street, Hsin-Yi District, Taipei 11031, Taiwan

Tel: 886-2-27372181 ext. 1131

e-mail: sandychen@tmu.edu.tw

Chao-Yuan Yeh, aetherAI Co., Ltd., 3-2, YuanQu Street, Nan-Gang District, Taipei 115, Taiwan

Tel: 886-2-27856892

e-mail: joeyeh@aetherai.com

* These authors contributed equally to this work.

** Correspondence and requests for materials should be addressed to C-Y Y. (joeyeh@aetherai.com) or C-Y C. (email: sandychen@tmu.edu.tw).

Supplementary Figures

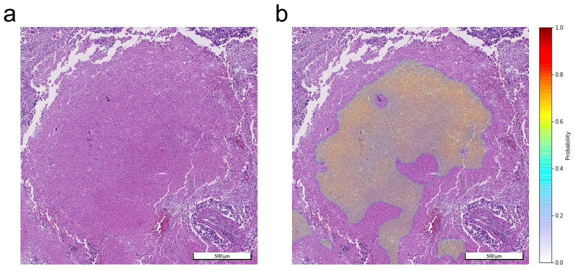

Supplementary Figure 1. Tissue necrosis highlighted by CAM. a, Tissue necrosis under microscope. **b**, Our method occasionally highlights necrosis as tumor area since the high correspondence between the presence of necrosis and squamous cell carcinoma. The color of the overlaid heat map represents the prediction score ranging from 0 (lowest) to 1 (highest), defined in the color bar.
