# Supplementary material for: An annotation-free whole-slide training approach to pathological classification of lung cancer types using deep learning
Source: Nat Commun. 2021 Feb 19;12:1193. doi: 10.1038/s41467-021-21467-y (PMC7896045; doi:10.1038/s41467-021-21467-y)
Supplement: Supplementary file 4 — Description of Additional Supplementary Files [file 41467_2021_21467_MOESM4_ESM.pdf]

Title: Supplementary Data 1.

Description: Raw data of models' predictions, learning curves and throughputs. The models' prediction score and ground truth for each slide in our dataset are recorded in the tabs "Test Results for Adeno" and "Test Results for Squamous", as the underlying data of Figure 2, Figure 5 and Table 1. The results for small lesion dataset, TCGA-diagnostic dataset and TCGA-tissue dataset are in the tabs "Small-lesion Test Results", "TCGA-diagnostic Test Results" and "TCGA-tissue Test Results" respectively, as the data of Figure 2 and Table 1. The tab "Learning curve" contains the raw data for Figure 3, and "Throughput" for Figure 6.
